# Droplets Self-Draining on the Horizontal Slippery Surface for Real-Time Anti-/De-Icing

**DOI:** 10.1007/s40820-025-01908-9

**Published:** 2025-09-08

**Authors:** Xiao Han, Xu Sun, Di Zhao, Mingjia Sun, Kesong Liu, Liping Heng, Lei Jiang

**Affiliations:** 1 State Key Laboratory of Bioinspired Interfacial Materials Science, School of Chemistry, Beihang University, Beijing, 100191 People’s Republic of China; 2 State Key Laboratory of Bioinspired Interfacial Materials Science, Bioinspired Science Innovation Center, Hangzhou International Innovation Institute, Beihang University, Hangzhou, 311115 People’s Republic of China

**Keywords:** Slippery surfaces, Droplet self-draining, Anti-/de-icing, Thermoelectric coupling, Charge transfer

## Abstract

Undesired ice accumulation on infrastructure and transportation systems leads to catastrophic events and significant economic losses. Although various anti-icing surfaces with photothermal effects can initially prevent icing, any thawy droplets remaining on the horizontal surface can quickly re-freezing once the light diminishes. To address these challenges, we have developed a self-draining slippery surface (SDSS) that enables the thawy droplets to self-remove on the horizontal surface, thereby facilitating real-time anti-icing with the aid of sunlight (100 mW cm^−2^). This is achieved by sandwiching a thin pyroelectric layer between slippery surface and photothermal film. Due to the synergy between the photothermal and pyroelectric layers, the SDSS not only maintains a high surface temperature of 19.8 ± 2.2 °C at the low temperature ( −20.0 ± 1.0 °C), but also generates amount of charge through thermoelectric coupling. Thus, as cold droplets dropped on the SDSS, electrostatic force pushes the droplets off the charged surface because of the charge transfer mechanism. Even if the surface freezes overnight, the ice can melt and drain off the SDSS within 10 min of exposure to sunlight at  −20.0 ± 1.0 °C, leaving a clean surface. This work provides a new perspective on the anti-icing system in the real-world environments.

**Supplementary Information:**

The online version contains supplementary material available at 10.1007/s40820-025-01908-9.

## Highlights

- Self-draining slippery surface with light-thermal-electric synergy were fabricated to auto anti/de-icing even on horizontal devices.
- The synergy of photothermal conversion and thermoelectric coupling enables the ice melting, and self-draining of thawy droplets at the same time, avoiding the risk of re-freezing.
- The processes of no matter in ice melting or droplets repulsion on horizontal surface need no additional energy input, just with assistance of sunlight.

**Supplementary Information:**

The online version contains supplementary material available at 10.1007/s40820-025-01908-9.

## Graphical abstract

**Figure:**
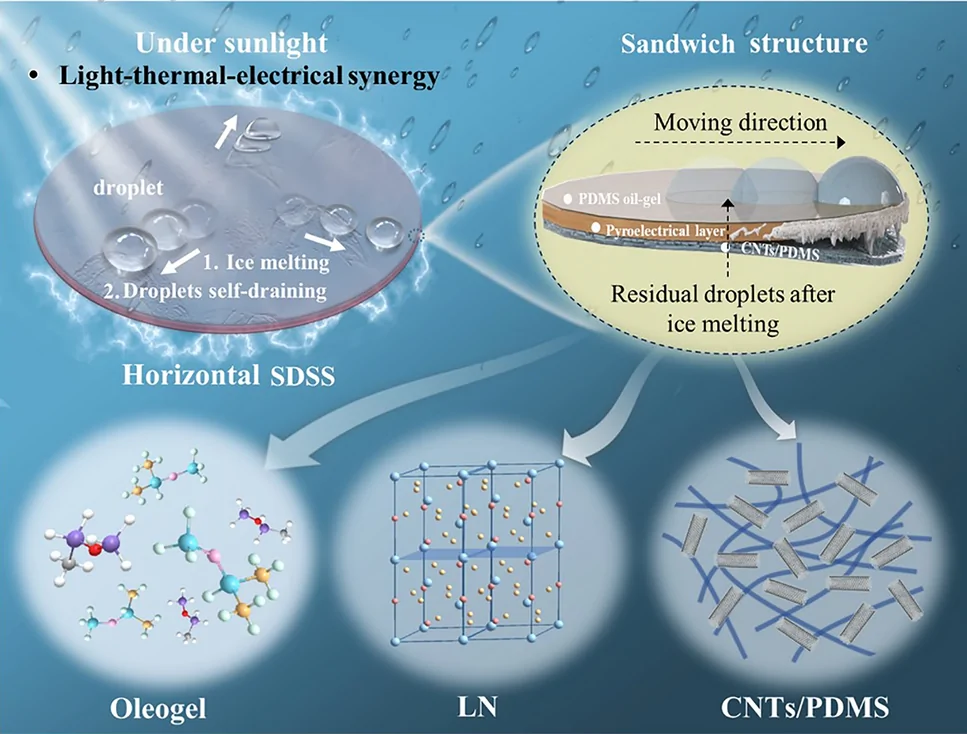

## Introduction

Icing is a common event at high latitudes, impeding the stable operation of various industries due to its accumulation on buildings and roads, ships and aircraft, antennas and meteorological instruments, power lines, and pipelines [1–4]. According to statistics, the global economic loss reached $10 billion due to extreme blizzards in 2024 [5]. In light of this, significant human and material resources are allocated to post-blizzard cleanup efforts to reduce the occurrence of accidents and mitigate substantial economic losses, including the implementation of mechanical de-icing and chemical de-icing procedures [6, 7]. However, such conventional active de-icing methods not only lack timeliness but also consume vast amounts of energy and chemicals (> 5 kW m^−2^), posing environmental and safety hazards [8, 9]. Therefore, developing auto anti-/de-icing materials that can prevent ice coverage or even realize ice self-cleaning is a pressing and effective approach.

Recently, suppression or reduction of icing using ice-phobic surfaces has been a hot topic [10–14]. Among them, anti-icing surfaces such as photothermal superhydrophobic surface (PSHS) [15–18] and photothermal slippery surface (PSS) [19] are the industrywide foundation to mitigate icing effect in a large variety of scenarios due to their exceptional water-repellent property [20, 21]. However, despite the common anti-icing capability of the PSHS, in high humidity environments, tiny droplets will first form within the micro-nanostructures of the supercooled PSHS and nucleate into ice crystals, thus gradually diminishing the effect of anti-icing [22, 23]. Crucially, the PSHS that lacks self-healing properties will suffer from the permanent damage of their superhydrophobicity, when subjected to physical damage [24]. On the other hand, further notable development of the PSS can overcome the above weaknesses of PSHS [25]. Due to the advantages of self-healing [23, 26, 27], humidity tolerance [28], and reduced freezing nucleation sites [29, 30], PSS has important application prospects in the field of anti-icing [31–33]. Nevertheless, even with photothermal effect, both PSHS and PSS can melt the ice layer on their surface, the resulting thawy droplets still remain on the horizontal surface without component of gravity [34, 35]. Particularly, this issue is usually pronounced for micro-/nano-scale condensate droplets formed in low-temperature, high humidity environments, on which the depressed gravity is even insufficient to overcome the surface adhesion. In other words, droplets that are not promptly removed from horizontal surfaces would undergo the re-freezing once the external field is removed, thereby hindering the establishment of the auto anti-/de-icing system [36, 37]. Furthermore, the low adhesion state is easily compromised during icing and melting cycles (Cassie-to-Wenzel state), leading to poor self-removal capacity even under gravitational influence (even at 90° tilt). Therefore, the timely and spontaneous removal of ice formations, whether they exist as small frost droplets or large ice layers, remains an urgent challenge that must be addressed in the development of effective anti-icing materials.

**Fig. 1:**
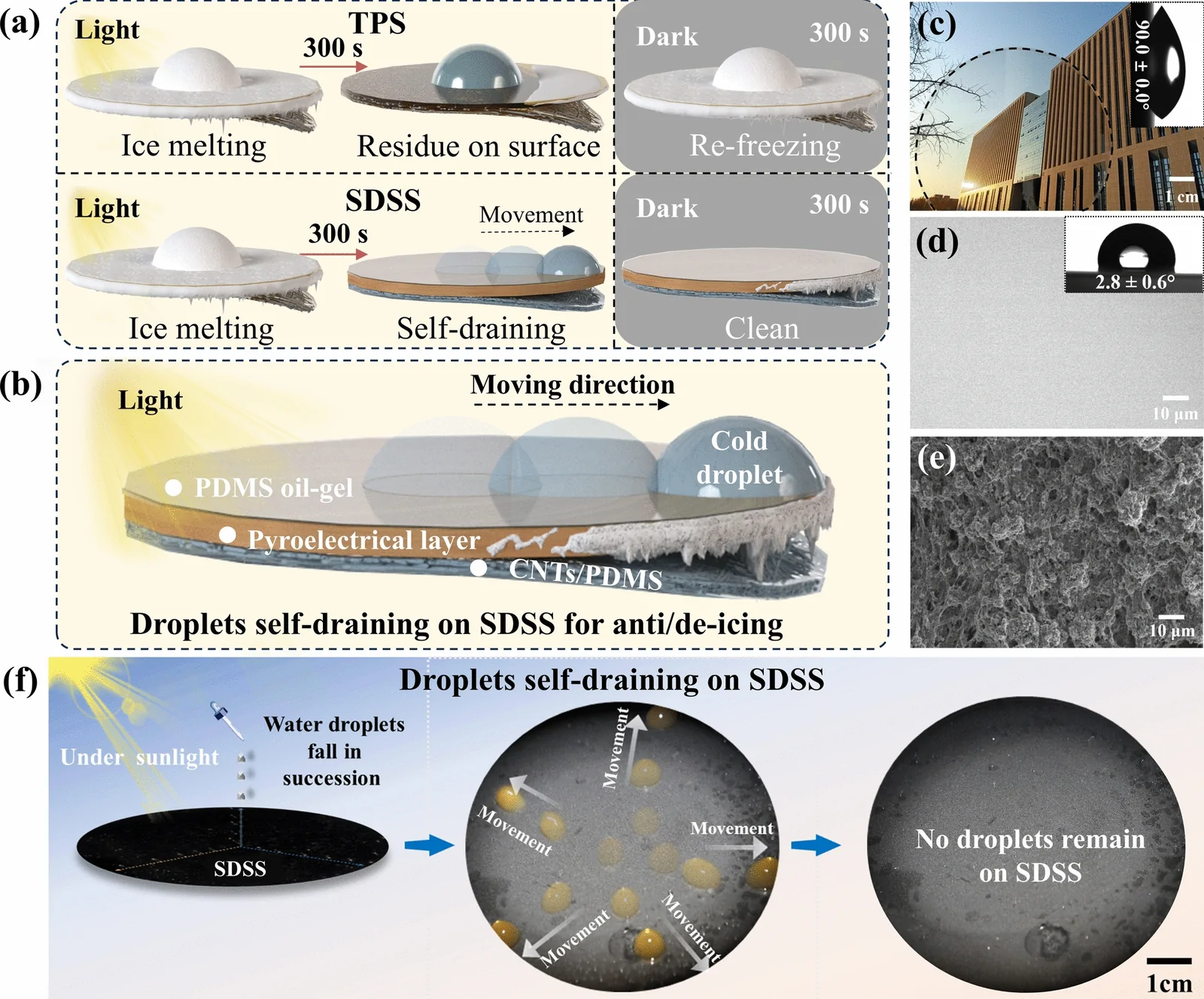
Design of the SDSS with the function of droplet self-draining. **a** Scheme of the icing process for traditional photothermal materials with residual droplets on its surface as the comparison with the SDSS.** b** Scheme of the droplet self-draining on the SDSS with sandwich structure of the top slippery surface, middle pyroelectric layer, and bottom photothermal layer. **c** Optical photo of LN with high transparency as the pyroelectric layer to provide the electrostatic force. The insert shows water droplet pinned on LN (5 μL). **d** Optical image of the silicone oil-infused PDMS as the top slippery surface. The insert shows the ultra-low sliding angle of the water droplet with only 2.8 ± 0.6° on slippery surface (5 μL). **e** SEM image of the CNTs/PDMS as photothermal layer to avoid the droplet from freezing. **f** Upon  100 mW cm^−2^ irradiation, the cold droplets (10 μL) that land on the horizontal SDSS surface spontaneously moving toward the edges, leaving behind a clean surface

Herein, a photothermal slippery surface with the self-expulsion behavior was fabricated by sandwiching a thin pyroelectric layer—such as lithium niobate (LN), polyvinylidene fluoride (PVDF), or lead zirconate titanate (PZT)—between a slippery film and a photothermal conversion (PC) layer. Due to the synergy between the photothermal and pyroelectric coupling, the self-draining slippery surface (SDSS) not only provides a high-temperature rising (39.8 ± 2.2 °C) with the assistance of sunlight, but also generates the electrostatic force spontaneously to push the droplets off the surface due to the local temperature gradient, preventing the secondary crystallization that typically occurs on the traditional photothermal surface (TPS, Fig. 1a). Specifically, under light irradiation (1 sun, 100 mW cm^−2^), the PC layer continuously provides thermal energy for SDSS to maintain a high temperature (19.8 ± 1.2 °C) at the ambient temperature of  −20.0 ± 1.0 °C, thus keeping the surface in an anti-icing state. Subsequently, the temperature gradient between SDSS and cold droplets (0.2 ± 0.5 °C) naturally induces the internal polarization of pyroelectric layer, which further triggers charge transfer between the droplets and the pyroelectric layer. Therefore, facilitated by the newly formed electrostatic repulsion and the low lateral adhesion (sliding angle < 5°) of the top slippery surface, the cold droplets can self-drain off the SDSS, leaving a clean surface as shown in Fig. 1a. The strategy offers a new insight into the fast-growing anti-icing technology and provides an approachable methodology to advance the development of a new generation of energy-efficient and smart de-icing systems.

## Experimental Section

### Materials

Polydimethylsiloxane (PDMS, Sylgard 184) and curing agent were obtained from Dow Corning, USA. N-hexane was purchased from Sigma-Aldrich Inc. Hydroxylated carbon nanotubes were purchased from Suzhou Tanfeng Graphene Technology Co. Ltd. Active carbon and carbon paint were purchased from Jing Ji Technology Co., Ltd. PVDF and PZT were purchased from Kosen Electronics Co., Ltd. LiNbO_3_ single crystals were purchased from Putian Antek Optics Co., Ltd. Silicone oil (20 cSt) was purchased from Beijing Zhongke Shangde Technology Co., Ltd. RhB and copper sulfate powders were purchased from Sinopharm Chemical Reagent Co., Ltd. Hexadecyl trimethyl ammonium bromide (CTAB) was purchased from Shanghai McLean Biochemical Technology Co., Ltd. Ethanol was purchased from Sinopharm Chemical Reagent Co., Ltd. All chemicals were used as received without further purification. All wetting and rinsing processes used deionized water with a resistivity of 18.2 MΩ cm (Milli-Q).

### Preparation of the SDSS

First, a 500 μm thick, 3-inch-diameter LN substrate was ultrasonically cleaned in deionized water and anhydrous ethanol for 15 min. Next, an oleogel layer was prepared on the one side of the LN. Specifically, PDMS monomer and its crosslinking agent were uniformly mixed at a weight ratio of 10:1, and diluted with an appropriate amount of n-hexane (PDMS: n-hexane = 5 wt%), followed by spin-coating onto LN (1000 r min^−1^, 30 s). Subsequently, in order to equip with the photothermal layer, PDMS monomer and its crosslinking agent were uniformly mixed at a weight ratio of 10:1, following by mixing with a certain amount of carbon nanotubes (50 wt%). Then, adding CTAB (mass ratios of 1:1 to PDMS), and an appropriate amount of n-hexane (PDMS: n-hexane = 5 wt%) to dilute to a suitable consistency. After ultrasonication for 60 min, the above mixed dispersion was spin-coated onto another side of LN. After curing at room temperature for 6 h, a layer of 20 cSt silicone oil (800 r min^−1^, 20 s) was spin-coated on the side of PDMS, followed by getting rid of the redundant silicone oil. In addition, the preparation of SDSS samples with PVDF or PZT as pyroelectric layer is consistent with the above method.

### Preparation of the TPS

First, 3-inch-diameter glass substrate was ultrasonically cleaned in deionized water and anhydrous ethanol for 15 min. Next, PDMS monomer and its crosslinking agent were uniformly mixed at a weight ratio of 10:1, following by mixing with carbon nanotubes at mass ratios of 1:1. Then, adding CTAB (mass ratios of 1:1 to PDMS) and an appropriate amount of n-hexane (PDMS:n-hexane = 5 wt%) to dilute to a suitable consistency. After ultrasonication for 60 min, the above mixed dispersion was spin-coated onto the glass. The TPS sample was obtained after curing for 6 h at room temperature and spin-coating layer of 20 cSt silicone oil.

### Basic Characterization

SEM images were obtained with an environmental FESEM (Quanta FEG250, FEI). All the CAs and SAs of droplets were measured using a contact angle meter (Dataphysics OCA25, Germany). The average value was obtained by measuring five different points on the sample surface. All of the contact angle and sliding angle measurements were carried out at 25.5 ± 1.0. The optical photos were recorded using a digital camera (Canon 60D, Japan). Before solution preparation, samples are weighed using an electronic balance (Sartorius GL623-1SCN, Germany). Infrared thermal images were obtained by thermal imager (Fluke Ti480 PRO, USA).

### Anti-icing Experiments

The test conditions of the anti-icing experiment were  −20.0 ± 1.0 °C and 80% relative humidity. In order to maintain a relatively stable environment, we use transparent PMMA to build a closed square chamber, in which a semiconductor cooler for controlling temperature and a humidifier for controlling humidity are placed to create a relatively stable closed environment. During the test, the test sample was placed horizontally on the semiconductor cooler. When the surface temperature of the sample was maintained at  −20.0 ± 1.0 °C, and the relative humidity of the indoor environment was controlled at 80%, the simulated light source was turned on to provide light, and the samples’ icing processes were observed and recorded.

### De-icing Experiments

The test conditions of the de-icing experiment were  −20.0 ± 1.0 °C and 80% relative humidity. Firstly, in the closed PMMA chamber, the test sample was placed on the semiconductor cooler at -20.0 ± 1.0 °C and 80% relative humidity for 2 h to form a uniform frost/ice layer on the sample surface. Then, turning on the xenon lamp light source and recording the frost/ice melting and exclusion. During the whole test process, the sample was always placed horizontally without any tilt angle or external force interference.

### Electrical Experiments

The surface potential under simulated sunlight was measured by an electrostatic voltmeter (Monroe model 244A, US), equipped with the home-made scanning table. After placing the SDSS below the light source (the diameter of the light source is ~ 4 cm), the surface potentials of the samples were measured at different temperature rises by controlling the light intensity. In addition, to verify the potential change after droplet dropping on the SDSS, the cold droplet (0 °C) was dropped on the central of the SDSS with temperature of 20 °C. Subsequently, in order to avoid the potential shielding of the droplet, the droplet was removed quickly, followed by measuring the surface potential of SDSS by electrostatic voltmeter. On the other hand, the amount of charge carried by the droplets was measured by a Faraday cup connected to an electrometer (Keithley 6514). The required volume of cold droplet (0 °C) was measured by a grounded micro-syringe, and then, the droplets are released at about 1 cm above the sample. The droplets fall into the Faraday cup after contact with the sample surface, and the amount of charge carried by the droplets after contact with the sample surface were measured.

### Adhesion Strength Experiments

First, fill a 10 mm × 10 mm × 30 mm cuvette to two-thirds of its volume with water. Subsequently, invert the cuvette onto the sample and transfer them into a cryogenic refrigerator at  −60 °C for 3 h. The SF-500 digital force gauge (Quzhou Aipu Measuring Instrument Co. Ltd.) was gently pushed on the frozen cuvette in the direction parallel to the sample surface, and the shear stress required to detach each ice column from the surface was obtained. Thus, the ice adhesion strength was obtained by dividing the adhesion force by the contact area.

## Results and Discussion

### Design Principle and Structural Characterizations

To achieve the droplet self-expulsion and thus anti-icing in the sub-zero conditions without applying any inclination angle as shown in Fig. 1a, we fabricated SDSS by stacking three homogeneous layers using a simple spin-coating technique (Fig. S1). As shown in Fig. 1b, SDSS consists of three layers: a slippery surface (PDMS oleogel, ~ 50 μm), a pyroelectric layer (LN, ~ 500 μm), and a PC layer (CNTs/PDMS, ~ 600 μm), respectively. Among that, to provide a horizontal electrostatic force for realizing the surface droplets self-draining, the transparent LN that can release the thermally induced charge was selected as pyroelectric layer as shown in Fig. 1c. However, the hydrophilic LN surface with great adhesion to water blocks movement of the droplets (water droplet pinned on LN even with tilting angle of 90.0°). Therefore, to reduce the viscous resistance of SDSS for removing surface droplets, the silica oil-infused oleogel was coated on one side of the pyroelectric layer as the slippery surface. After modification, the slippery surface is still flat and smooth, on which the sliding angle drops sharply to 2.8 ± 0.6° (Fig. 1d). On the another side, to prevent water on the slippery surface from freezing at low temperatures, the CNTs/PDMS films with high photothermal conversion properties were deposited on the back of the pyroelectrical layer (Fig. 1e). As shown in Fig. S2, CNTs slurry with partial micron-sized clusters is uniformly adhered on the pyroelectrical surface by crosslinked PDMS. Thus, the combination of above functional materials can efficiently achieve droplet self-draining on the horizontal SDSS without inclining the surface or applying external disturbances. Compared of the traditional photothermal slippery surface and superhydrophobic surface, the ice water (10 μL), sequentially dropped on the heated SDSS, would move spontaneously toward the edge under sunlight (Fig. 1f).

### Mechanism of the Droplet Self-draining on Horizontal SDSS

**Fig. 2:**
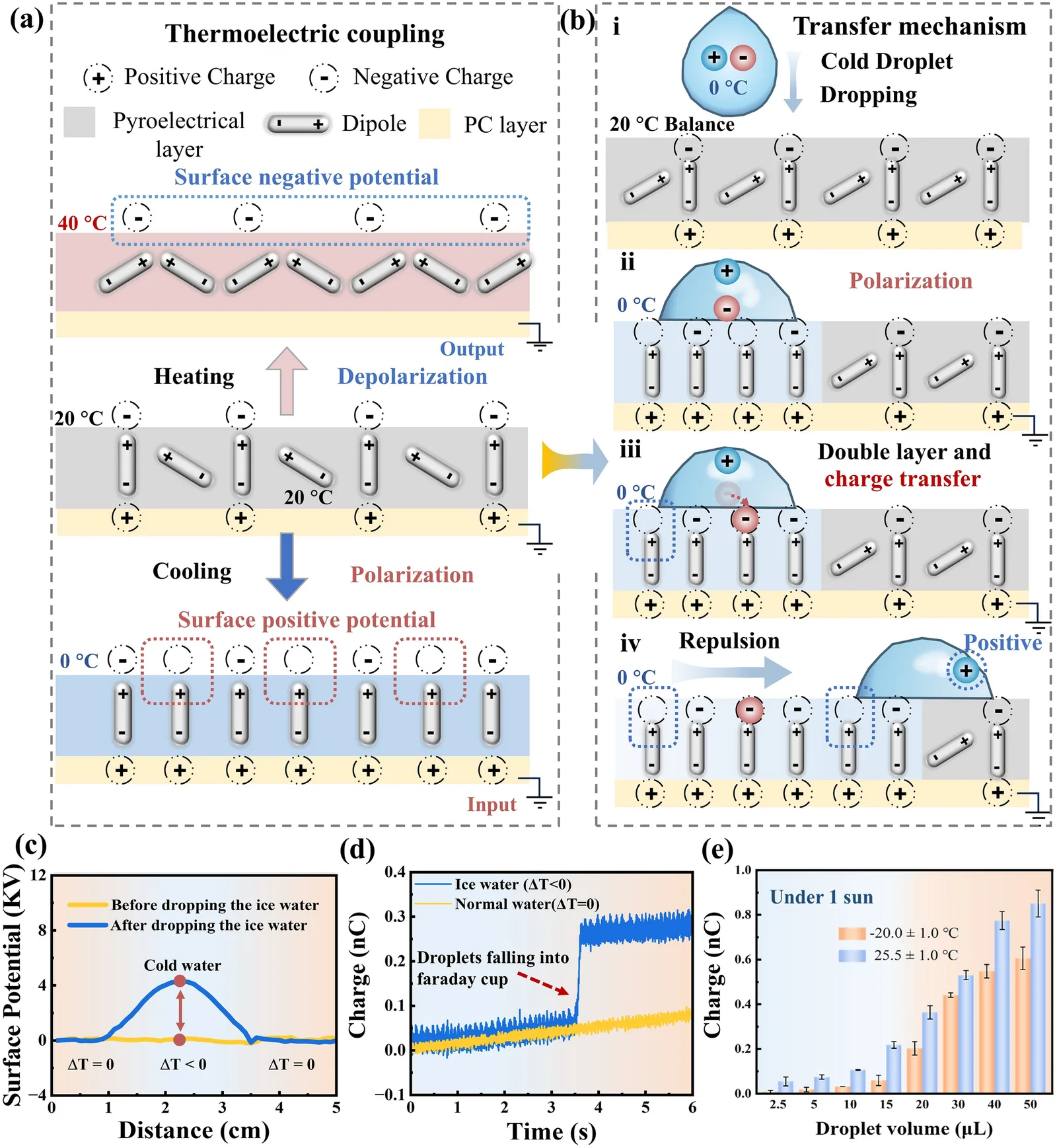
Mechanism of the droplet self-draining on SDSS. **a** Thermoelectric coupling process of the pyroelectrical layer. When subjected to temperature changes, the dipoles within the material align or lose their alignment, contributing to the piezoelectric effect that generates electric charge in response to the thermal changes. **b** Mechanism of the cold droplets self-draining on the SDSS. As cold droplets (0 °C) come into contact with the SDSS surface at room temperature (20 °C), the temperature difference causes dipole orientation to form a double electric layer with the negative charges in the droplets, leading to partial charge transfer. Subsequently, electrostatic repulsion formed between SDSS and droplets with positive charges enables the cold droplets to self-remove on the SDSS. **c** Distribution of the surface potential before and after cold droplet (0 °C) dropping on the SDSS with temperature of 25.5 °C. **d** Comparison of the charge carried by room temperature droplets (25.5 °C) and cold droplets (0 °C) after moving on the SDSS surface with temperature of 25.5 °C. **e** Relationship between droplet volume and amount of charge on droplets at different ambient temperatures

To understand why cold droplets can self-removal on the horizontal SDSS, we propose the thermoelectric coupling and charge transfer mechanism. As a typical pyroelectric material, LN in SDSS has the obvious thermoelectric coupling effect (Fig. 2a). Due to the exceptionally high Curie temperature (~ 1214 °C), LN still retains partially polarized dipoles at room temperature (20 °C), on which the free charges in the air will be attracted to form the double layer and maintain charge conservation [38, 39]. Therefore, when SDSS is heated up (∆T > 0), pyroelectric layer will depolarize, in which the orientation of dipoles is random. Thus, the surface negative charge on SDSS is released, resulting in the decrease of the surface potential. Correspondingly, if the SDSS cools down (∆T < 0), the pyroelectric layer will re-polarize. Due to the grounded PC layer, the negative part of the oriented dipole can be shield. Therefore, the positive part of the oriented dipole forms the hole, causing the whole SDSS with the positive potential. Similarly, if the ice water (0 °C) is dropped on the SDSS at room temperature (20 °C) as shown in Fig. 2b, the transfer of temperature will cause the local dipole to re-orient. In this process, it is believed that partial dipoles can easily form a double layer with negative charges in the cold droplet at the contact area, which results in the subsequent partial charge transfer [40, 41]. It is believed that trigger condition of the contact electrification (CE) is the double-layer theory at the solid–liquid interface [42, 43]. As a result, due to the partial charge transfer, the repulsive electrical force provided by SDSS pushes the droplets moving continuously to the edge.

To verify above-mentioned charge transfer theory, we measured the potential distribution of the SDSS after a cold droplet dropped on. Compared to the region without droplet, the strong spontaneous polarization occurs in the region with heat transfer, forming a non-uniform potential gradient that decays around the center of the droplet area. Of note, before measuring the potential, the residual charge on the sample surface has been removed by an ionizing blower. Thus, the obvious generation of surface potential on SDSS (4.22 ± 0.37 kV) can prove strongly its sensitivity of the thermoelectric coupling (Fig. 2c). Besides, as the cold liquid droplets move, the temperature and potential distributions on the SDSS surface undergo corresponding changes, with their distribution curves remaining largely consistent (Note S2, Fig. S3). That is to say, as the droplet migrates, region of the surface potential gradually expands and the transferred charge accumulates, which causes the increased droplet velocity (Note S3, Fig. S4). Therefore, on the other hand, the carried charge by droplet after self-draining to the edge was also measured by Faraday Cup with electrometer. Compared with droplets at ambient temperature, the ice water displayed a distinct electrical signal step (0.22 ± 0.05 nC) at the moment it fell into the Faraday Cup (Fig. 2d). The contrast phenomenon proves the charge transfer mechanism between cold droplets and LN, which is caused by natural temperature difference rather than triboelectricity [44, 45]. Therefore, the cold droplets with positive charge can be repelled by SDSS with oriented dipole, proving the synergistic effect of thermoelectric coupling and charge transfer. More importantly, apart from the LN with single crystal, other pyroelectrical materials, no matter in terms of organic PVDF, or inorganic PZT, can also realize the droplet self-draining as shown in Fig. S5, proving the universality of the mechanism. Moreover, surface charge transfer occurs not only in cryogenic liquids; for hot droplets exhibiting a temperature difference with SDSS, the associated charge transfer mechanism and resultant electrostatic repulsion remain operative as well (Note S4, Fig. S6).

**Fig. 3:**
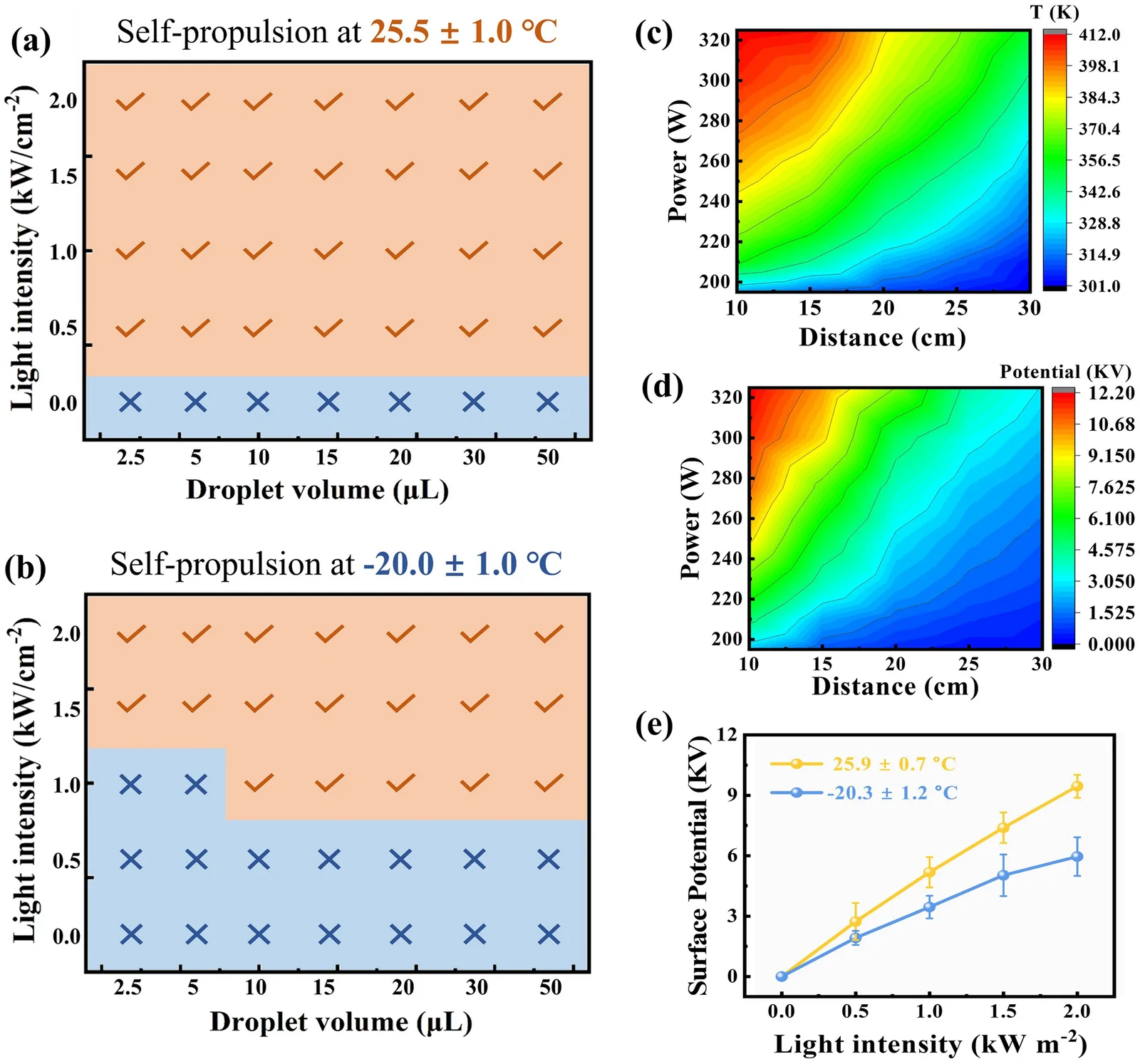
Factors for influencing droplet self-draining. **a, b** Phase diagram of droplets (0 °C) self-propulsion on SDSS as the functions of droplet volume and light intensity at ambient temperature of 25.5 °C (a) and  −20.0 °C (b). **c** Temperature rising of the SDSS influenced by light power and illumination distance. **d** Surface potential of the SDSS influenced by light power and illumination distance. **e** Plot of surface potential of the SDSS vs light intensity at different ambient temperatures

In addition, we found that the larger the droplet volume is, the more amount of charge is carried by droplets due to the larger contact area with surface as shown in Fig. 2e. Therefore, the bigger droplet is more easily repelled and cleaned due to the stronger electrostatic repulsion (Figs. 3a and S7). Moreover, not only can deionized water achieve self-removal on the SDSS surface, but this phenomenon is also attainable with acidic solutions, alkaline solutions, or solution with ions (Fig. S8). With increasing ionic concentration and corresponding conductivity of the liquid, the surface free charges released from LN depolarization are more easily transferred to the ion-containing liquid (Fig. S9). It is worth noting that, the increased amount of transferred charge is negligible compared to the generated charge on the SDSS with local cold area. According to relationship between charge density (*ρ*) and temperature difference (Δ*T*) [46],

1 $$\rho = P_{{\mathrm{c}}} \Delta T$$

the released charge density by SDSS can reach about 80 nC cm^−2^ (∆*T* = 20 °C), due to the high pyroelectric coefficient* P*_c_ of the lithium niobate (~ 4 × 10^–5^ C m^−2^ k^−1^). In this case, when a supercooled droplet (0 °C, 10 μL) dropped on the SDSS (contact area of 28 mm^2^), the generated charge (*Q* = *ρS*) can be calculated as about 25.6 nC; whereas, the amount of transferred charge does not even exceed 0.5 nC (Fig. S10). Thus, as increased transferred charge on solution with ions, the powerful electrostatic repulsion elevates the droplet’s velocity from 5 to 22 mm s⁻^1^ (0–1 M NaCl solutions). In addition to properties of the droplet, the ambient temperature also affects the charged amount of the droplets (Fig. 2e). Even under the same conditions, the transferred charge of droplets in ambient environment of 25.5 °C is 1.2–1.8 times that in low temperature ( −20.0 °C). Thus, with assistance of 0.5 sun, all volume of the droplets (2.5−50 μL) can be cleaned without any external field when ambient temperature is 25.5 °C (Fig. 3a). But when the ambient temperature is reduced to  −20.0 °C, only droplets with a volume of more than 10 μL can self-drain with the assistance of 1 sun (Fig. 3b). Although the droplet self-propulsion gets similarly easier as enhanced light intensity and droplet volume, the driving threshold at different ambient temperatures is not consistent. Because even under the same light intensity, the temperature rise in different environments is not consistent (Fig. S11), which greatly affects the polarization degree and the surface potential. It is believed that the surface potential (*E*) on SDSS presents a positive correlation with the temperature rise (Δ*T*) [47]:

2 $$E = LP_{{\mathrm{c}}} \Delta T/\varepsilon_{0} \varepsilon_{{\mathrm{p}}}$$

where *L* and *ε*_p_ denote the thickness and permittivity of the lithium niobate, respectively, and *ε*_0_ is the dielectric constant of empty space. Besides, when the material reaches a steady state (heat generation = heat loss) under the irradiation, the temperature rise can be affected by [48]:

3 $$\Delta T = \eta I/h$$

where *η* is photothermal conversion efficiency, *I* is light intensity, and *h* is heat dissipation. Therefore, the relationship between the surface potential (*E*) and light intensity (*I*) is derived as:

4 $$E = \eta ILP_{{\mathrm{c}}} /h\varepsilon_{0} \varepsilon_{{\mathrm{p}}}$$

Thus according to Eqs. (3) and (4), due to the higher heat dissipation (*h*) for SDSS at the lower temperature, the corresponding lower surface potential at  −20.0 °C makes it more difficult to push droplets to move (Note S1). Fortunately, the surface potential of SDSS caused by temperature rise (∆*T*) is in direct proportion to the light intensity and photothermal conversion of materials. Therefore, to further improve the temperature rise of the SDSS and surface potential (*E*) at cold environment ( −20.0 °C), research on the light intensity and photothermal conversion property of SDSS is of great significance. Firstly, the surface temperature can be enhanced with the stronger light intensity, which is affected by power of the light source and the illumination distance (Fig. 3c). Under a series of light intensities from 0.5 to 2.0 kW m^−2^, the temperature rise (Δ*T*) of the SDSS gradually increased from 23.1 to 64.5 °C. Correspondingly, the measured surface potential is also proportional to the power of the light source and negative correlation with illumination distance, which can even reach 12 kV under irradiation of 320 W, and distance of 1 cm (Fig. 3d). In general, the measured surface potential can be adjusted by light intensity (Fig. 3e), although the increase rate at room temperature is higher than that at low temperature. Therefore, as the solar irradiance increases, the higher temperature difference and enhanced electrostatic driving force acting on the droplet. As shown in Fig. S12, when the SDSS is exposed to a light intensity greater than 40 W cm⁻^2^, droplets with a volume of 10 μL can be spontaneously removed from the surface. Besides, even under solar irradiation with oblique-angle, the SDSS surfaces maintain the effective expulsion capability for supercooled liquid droplets (Fig. S13). It is believed that the temperature and corresponding potential gradients facilitate the movement of droplets to some extent (Note S5).

### Photothermal Properties and Liquid Repellency Properties

**Fig. 4:**
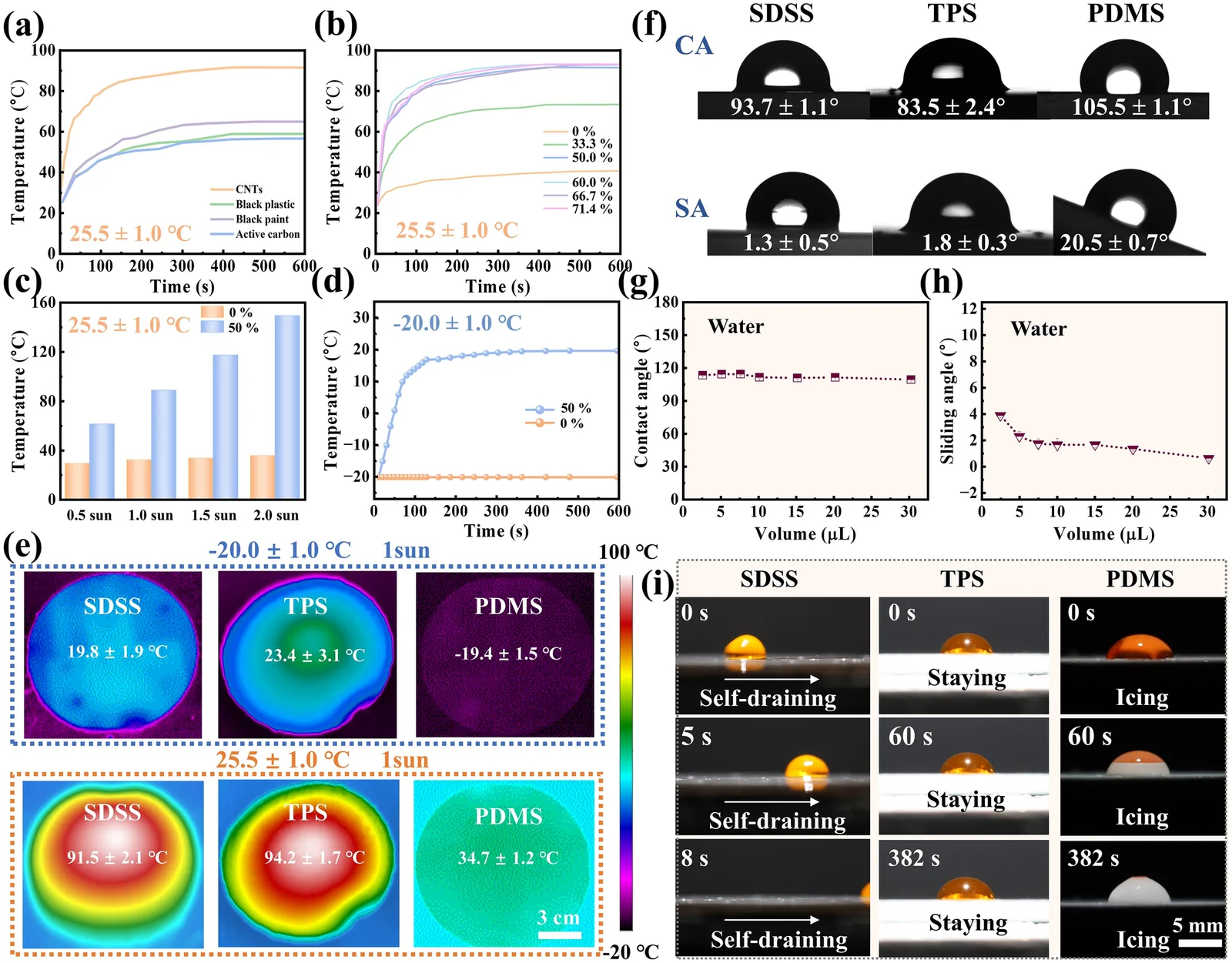
Photothermal properties and liquid repellency properties. **a** Temperature curves for using different PC nanomaterials with content of 50 wt% under 1 sun, such as carbon nanotubes, black plastic, black paint, and activated carbon. **b** Temperature curves of the PC films with different weight ratios of CNTs to PDMS under 1 sun illumination. **c** Surface stable temperature of SDSS and PDMS under different light intensities at 25.5 ± 1.0 °C. **d** Temperature of SDSS and PDMS varies with time under 1 sun illumination at  −20.0 ± 1.0 °C. **e** The optical images and infrared thermal images of SDSS, TPS, and PDMS under 1 sun illumination at  −20.0 ± 1.0 °C and 25.5 ± 1.0 °C. **f** SAs and CAs of 10 μL water on SDSS, TPS, and PDMS.** g** CAs change of water droplets with different volumes on SDSS surface. **h** SAs change of water droplets with different volumes on SDSS surface. **i** Comparison of dynamic process after droplets falling on the SDSS, TPS, and PDMS surfaces under 1 sun at ambient temperature of  −20.0 °C

In addition to light conditions, the basic photothermal conversion performance of the SDSS is the essential of the temperature rise of SDSS. Among that, in order to allow the light to pass through the pyroelectrical layer and reach the underlying PC layer, the near-transparent LN with a light transmittance of 0.88 was selected from the numerous pyroelectric materials [49] as a transparent window (Fig. S14). The SDSS with transparent LN exhibits significantly higher photothermal conversion performance, achieving a temperature rise of up to 64.5 °C under 1 sun illumination. In contrast, SDSS devices with non-transparent pyroelectric materials such as PZT (T-SDSS, light transmittance of 0.12) or PVDF (V-SDSS, light transmittance of 0.45) only reach a temperature rise of approximately 10.2 and 26.7 °C under the same illumination conditions (Fig. S15). Therefore, to enhance the photothermal de-icing efficiency, the transparency of the photothermal conversion layer and the top slippery surface should be guaranteed. On the other hand, we systematically studied the photothermal properties of common PC nanomaterials, mainly including CNTs, black plastic, black paint, and activated carbon (Fig. 4a). Among them, CNTs exhibit the maximum temperature rise under the same conditions, which even reaches 90 °C under illumination of 1 sun at 25.5 °C. Moreover, the photothermal property is closely related to addition amount of PC nanomaterials. For example, with irradiation of sunlight (100 mW cm^−2^), the stable surface temperature increases with addition of the CNTs (Fig. 4b). Yet as soon as the content exceeds 50 wt%, change of the temperature rise is not obvious as the extra addition (50−71.6 wt%), fluctuating at 64.3 °C (∆T). Likewise, once the thickness of PC layer reaches ~ 600 μm, the increase in thickness cannot further improve the photothermal performance significantly (Fig. S16). Thereby, the PC layer with thickness of 600 μm and 50 wt% CNTs content is selected for next study. Compared with PDMS without PC layer as shown in Fig. 4c, SDSS exhibits the distinct temperature rise to avoid the icing. Specifically, under the light intensity from 0.5 to 2.0 kW m^−2^, the stable temperature of the SDSS surface is 63.9, 91.5, 120.1, and 152.4 °C, respectively, showing the much higher surface temperature than that of the PDMS surface at room temperature (25.5 °C). Even at low-temperature environment ( −20.0 ± 1.0 °C), surface temperature of the SDSS and TPS still reaches 19.8 ± 1.9 °C and 23.4  ±  3.1 °C under 1 sun illumination, respectively (Fig. 4d, e). It is believed that such high-temperature rise is sufficient to prevent icing, polarize the pyroelectrical layer, and thus generate enough electrostatic force for cold droplet to self-remove (Fig. S17).

Yet to drive the cold droplets on the horizontal surface better, on the other hand, apart from providing the powerful external force, reducing the adhesion between droplet and surface is of great vital. Thus, equipped with oil-infused slippery surface, SDSS exhibit the ultra-low viscous resistance. The contact angles (CAs) and sliding angles (SA) of droplets with 10 μL on the SDSS are 106.7 ± 1.1° and 1.3 ± 0.5°, respectively (Fig. 4f). Similarly, CA and SA of droplets with 10 μL on the TPS are 83.5 ± 2.4° and 1.8 ± 0.3°, respectively. As comparison, the SAs are 20.5 ± 0.7° on PDMS, indicating a larger adhesion to droplets. In addition, the ultra-low SAs (< 5°) of different volume droplets on SDSS illustrate that both large and small droplets can easily slide off the slippery surface (Figs. 4g-h and S18). The SDSS with slippery surface can sharply reduce the surface adhesion and guarantee a frictionless motion of liquid droplets. Therefore, under sunlight irradiation, the energy conversion leads to the construction of an electrostatic field, enabling the cold droplets to self-roll off the horizontal SDSS and leaving a drip-free surface, as shown in Fig. 4i. As comparison, although the TPS exhibits an excellent anti-icing property, the thawy droplet that remains on the horizontal surface would freeze immediately as removal of the light field, just like the PDMS surface without PC layer (Fig. 4i).

### Anti-Icing and De-Icing Performance of SDSS

**Fig. 5:**
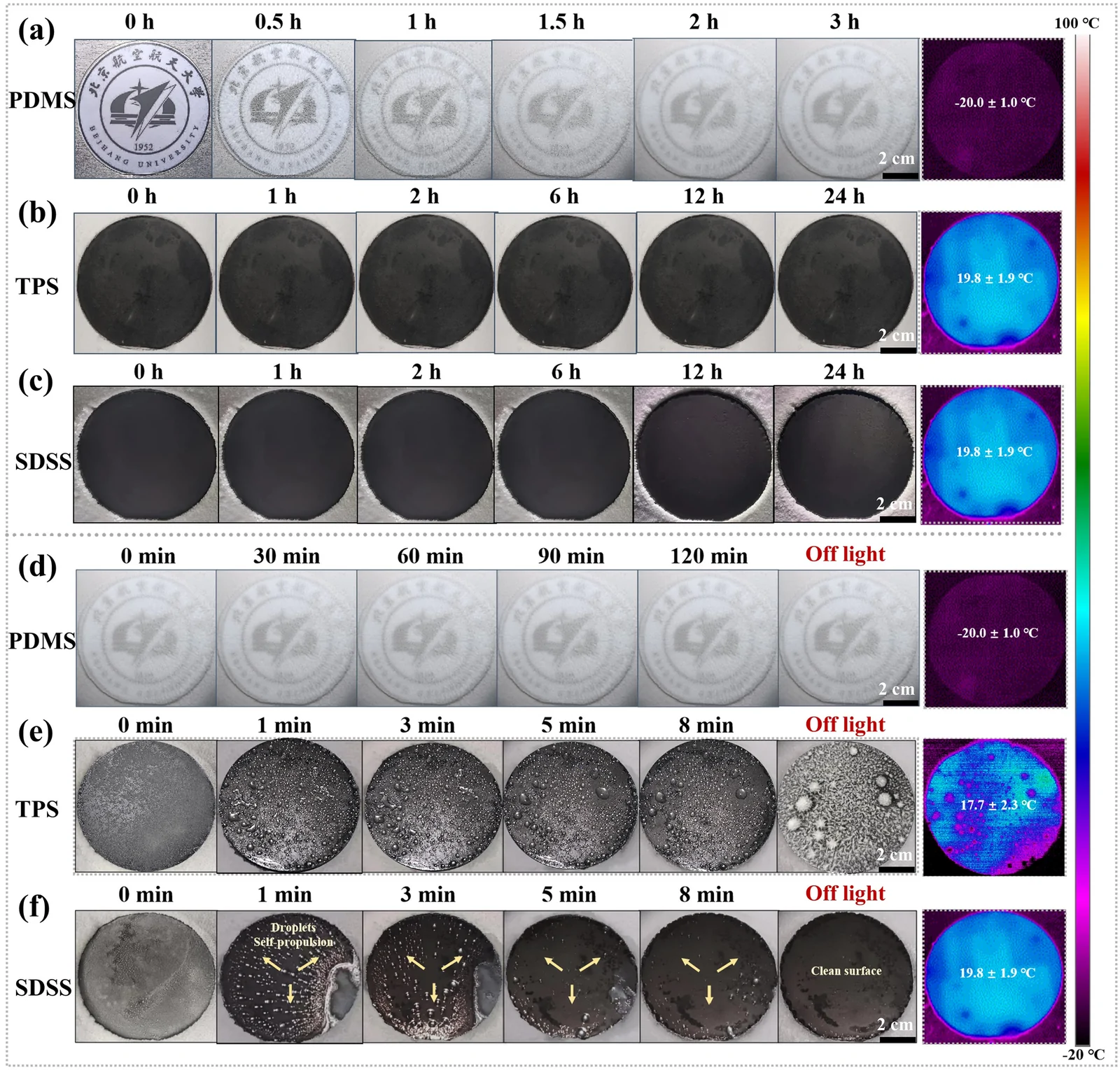
Anti-icing and de-icing performance. A series images illustrate the anti-icing performance of **a** PDMS, **b** TPS, and **c** SDSS under 1 sun illumination. Optical images showing the de-icing process of** d** PDMS,** e** TPS, and** f** SDSS under 1 sun illumination. The corresponding infrared images reflect the surface temperature of various materials at 1 sun. Experimental conditions: Temperature is  −20.0 ± 1.0 °C, and the relative humidity is 80%

To further illustrate the anti-icing and de-icing performance of SDSS at low-temperature environments, we compared the icing phenomenon of SDSS, TPS, and ordinary PDMS under conditions of  −20.0 ± 1.0 °C and 80% relative humidity. As shown in Fig. 5a, in the absence of a PC layer, the surface temperature of PDMS remains consistent with the ambient environment, resulting in the gradual accumulation of a thick ice layer within ~ 0.5 h, even under sunlight. In contrast, due to addition of the PC layer, both TPS and SDSS surfaces remain ice-free even after 24 h at low temperature ( −20 °C), maintaining a surface temperature of about 19.8 ± 1.9 °C (Fig. 5b, c). On the other hand, the de-icing capabilities are also the key indicator to validate the actual application value for materials. Specifically, all of samples were first frozen at  −20 °C and 80% RH in the dark for 2 h to perform an initial ice layer (Fig. S19). Subsequently, the samples were exposed to the light intensity of 100 mW cm^−2^ to access their de-icing performance. It was observed that the ice layer on the PDMS surface remained intact even after 180 min (Fig. 5d). As for TPS, although the ice layer on its surface could melt due to the photothermal effect of PC layer, the melted droplets remain on the horizontal surface. The residual droplets not only reduced the photothermal performance of the TPS to a certain extent (17.7 ± 2.3 °C), but also rapidly undergone the re-freezing within just 5 min after the sunset (Fig. 5e). Therefore, the TPS failed to establish a stable and effective anti-icing system. As a contrast, the SDSS could enhance effectively the surface temperature to 19.8 ± 1.9 °C in a short time and thus melt ice layer. Besides, due to thermoelectric coupling and charge transfer mechanism, newly established electrostatic field enabled the droplets to self-remove on the horizontal surface, leaving a clean surface within 8 min (Fig. 5f).

Moreover, the SDSS exhibits the exceptional operational stability in practical applications. Even after being placed in the normal environment for over 40 days, the liquid repellency of SDSS is basically consistent, as shown in Fig. S20. Due to the presence of PDMS, the infused silicone oil is firmly locked, effectively inhibiting its volatilization. This property allows SDSS to withstand prolonged water flow impact without degradation. For example, after being subjected to a water flow rate of 10 m s^−1^, the SA of droplets on SDSS only changes about 4° after 120 min, indicating strong resistance to water flow impacts and potential resilience against freezing rain (~ 5 m s^−1^). To further assess de-icing stability, we measured the adhesion strength between the SDSS and ice column across 100 repeated freeze–thaw cycles. As shown in Fig. S21, the adhesion strength of ice on SDSS during the 100 repeated freezing/thawing cycles almost has no obvious deterioration, exhibiting exceptional durability and environmental suitability of the SDSS. This long-term durability enables SDSS to function effectively under harsh conditions, such as high humidity (80%), sustained water flow impact (up to 180 min), and repeated icing–de-icing cycles (100 cycles), making it well-suited for demanding applications.

## Conclusions

In summary, the SDSS (combined with PC layer, pyroelectrical layer, and slippery surface) with character of the droplet self-draining was fabricated to enable anti-/de-icing (ambient temperature of  −20.0 ± 1.0 °C) even without any inclination. Under sunlight, the PC layer melts ice via the photothermal conversion and creates a temperature difference for pyroelectrical layer. This temperature difference, through thermoelectric coupling and charge transfer, generates a strong electrostatic field that charges cold droplets on the SDSS, synchronously forming electrostatic repulsion to drive droplets off the horizontal surface. The overlayer of slippery surface significantly reduces droplet adhesion (SA < 5°), promoting the removal of droplets and ice layer. Unlike traditional photothermal surface, the SDSS can self-clear the surface ice layer on the horizontal interface in time to remain a clean surface, thus preventing secondary icing of droplets even after the light fades. In addition, the environmental stability of the SDSS surface is also excellent. This work will provide new insights into manufacturing stable horizontal icephobic surfaces with superior anti-icing/de-icing properties for practical applications.

## Supplementary Information

Below is the link to the electronic supplementary material.

Supplementary file1 (DOCX 5933 KB)
