# Supplementary material for: Droplets Self-Draining on the Horizontal Slippery Surface for Real-Time Anti-/De-Icing
Source: Nanomicro Lett. 2025 Sep 8;18:60. doi: 10.1007/s40820-025-01908-9 (PMC12420567; doi:10.1007/s40820-025-01908-9)
Supplement: Supplementary file 1 — Supplementary file1 (DOCX 5933 KB) [file 40820_2025_1908_MOESM1_ESM.docx]

Supporting Information for

**Droplets Self-Draining on the Horizontal Slippery Surface for Real-Time Anti/De-Icing**

Xiao Han^1, 2^, Xu Sun^1^, Di Zhao^1^, Mingjia Sun^1^, Kesong Liu^1, 2^, Liping Heng^1,^ * and Lei Jiang^1^

^1^ State Key Laboratory of Bioinspired Interfacial Materials Science, School of Chemistry, Beihang University, Beijing 100191, P. R. China

^2^ State Key Laboratory of Bioinspired Interfacial Materials Science, Bioinspired Science Innovation Center, Hangzhou International Innovation Institute, Beihang University, Hangzhou 311115, P. R. China

*Corresponding author. E-mail: [henglp@buaa.edu.cn](mailto:henglp@buaa.edu.cn) (Liping Heng)

**Note S1 Driving force of droplet self-draining on horizontal SDSS**

Attributed to temperature change of the SDSS under light illumination, the released free charge on the pyroelectrical materials causes the particle charge transfer between SDSS and cold droplets. Subsequently, the newly formed electrostatic repulsion pushes the naturally charged droplets off the horizontal SDSS. Among that, the transferred charge on the droplet (Q) can be measured by Faraday cup. Thus the electrostatic force can be approximated as *F*_n_ *= EQ*, where the E is the electric field strength on SDSS, Q is the charge on droplet*.* In addition, It is believed that the surface charge density (ρ) released by pyroelectric layer presents a positive correlation with the temperature change (ΔT):

$$\rho=P_{\text{c}}\cdot|\Delta T|$$

where the *P*_c_ is pyroelectric coefficient. When the material reaches a steady state (heat generation = heat loss) under the irradiation, the temperature rise can be affected by:

$$\Delta T=\eta I/h$$

where η is photothermal conversion efficiency, I is light intensity, and h is heat dissipation. Thus the released charge density reads:

$$\rho=\eta IP_{\text{c}}/h$$

Subsequently, according to relationship between surface potential and charge density, the electric field strength can be described as:

$$E=\eta ILP_{\text{c}}/h\varepsilon_{0}\varepsilon_{p}$$

L and ε_p_ denote the thickness and permittivity of the lithium niobate, respectively, ε_0_ is the dielectric constant of empty space. Thus the electrostatic force becomes:

$$F_{n}=\eta ILP_{\text{c}}Q/h\varepsilon_{0}\varepsilon_{p}$$

According to the above formulas, the self-driving force for cold droplets on SDSS can be adjusted by pyroelectric coefficient, light intensity, photothermal conversion efficiency and heat dissipation. Among that, in order to allow the light to pass through the pyroelectrical layer and reach the underlying photothermal layer, the near-transparent LN with a light transmittance of 0.88, and pyroelectric coefficient of -4 ×10^-5^ C⋅K^-1^⋅m^-2^ was selected. Therefore, the capacity of the ice removal can be further improved by improving the light intensity parameters and the photothermal conversion performance of the photothermal materials.


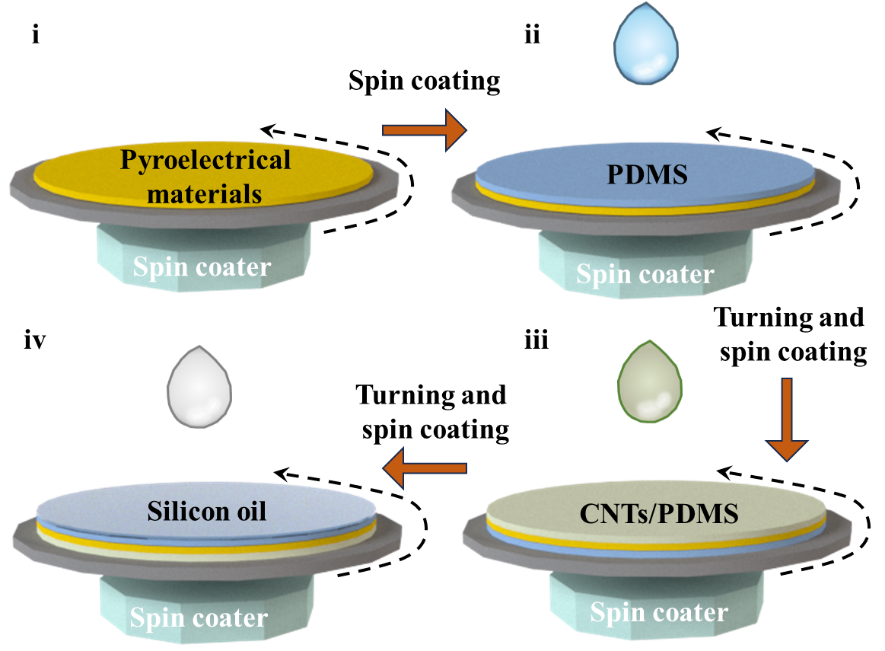


**Fig. S1** Preparation schematic of the SDSS with the sandwich structure


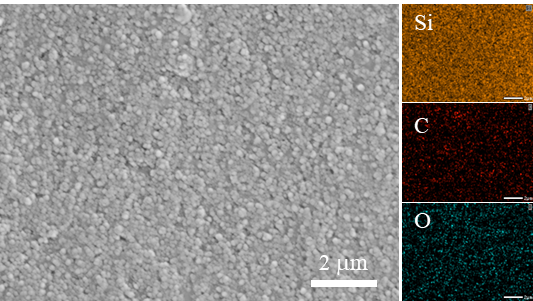


**Fig. S2** SEM and EDS mapping of the PDMS with photothermal CNTs


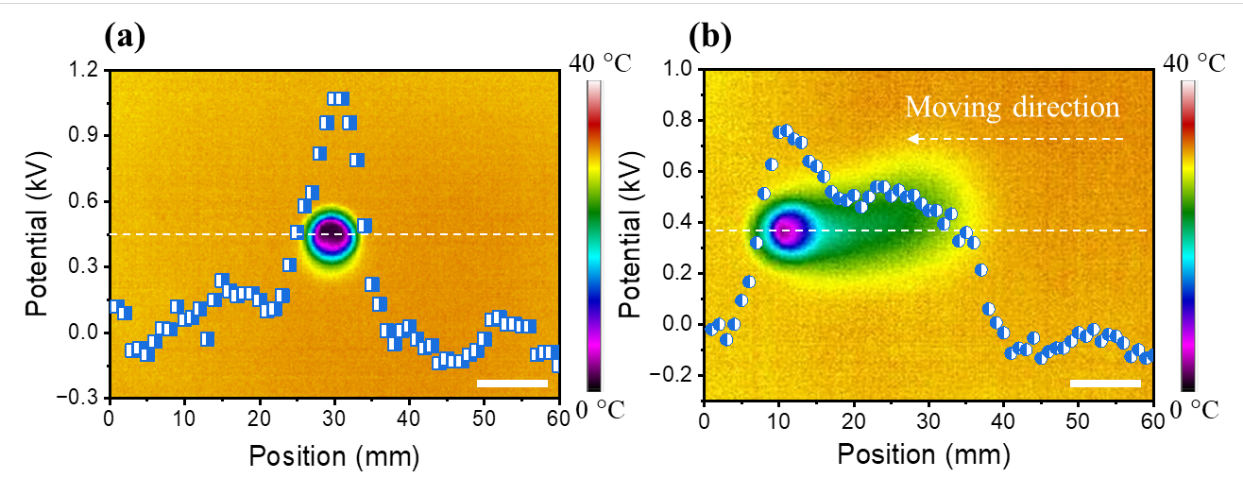


**Fig. S3** Potential distribution and corresponding temperature distribution on the SDSS before **a** and after **b** cold droplet self-propulsion. Scale bar, 10 mm

**Note S2 Potential distribution as droplet self-propulsion**

Due to the natural temperature difference between droplets and SDSS, the distribution of surface potential can self-adjust with the movement of the droplets to continuously generate repulsive force for liquid drops. Although the droplet temperature gradually increased as its self-propulsion (e.g. 0 - 5 °C), resulting in the lower electrostatic force and thus decreased transport speed from 8.9 ± 0.8 mm s^-1^ to 3.6 ± 1.5 mm s^-1^, the distance of droplet self-expulsion still reaches above 50 mm. In addition, with improvement of the photothermal conversion performance and thus the temperature difference (above 40 °C), the self-driving distance of melted ice layer is expected to be further improved.


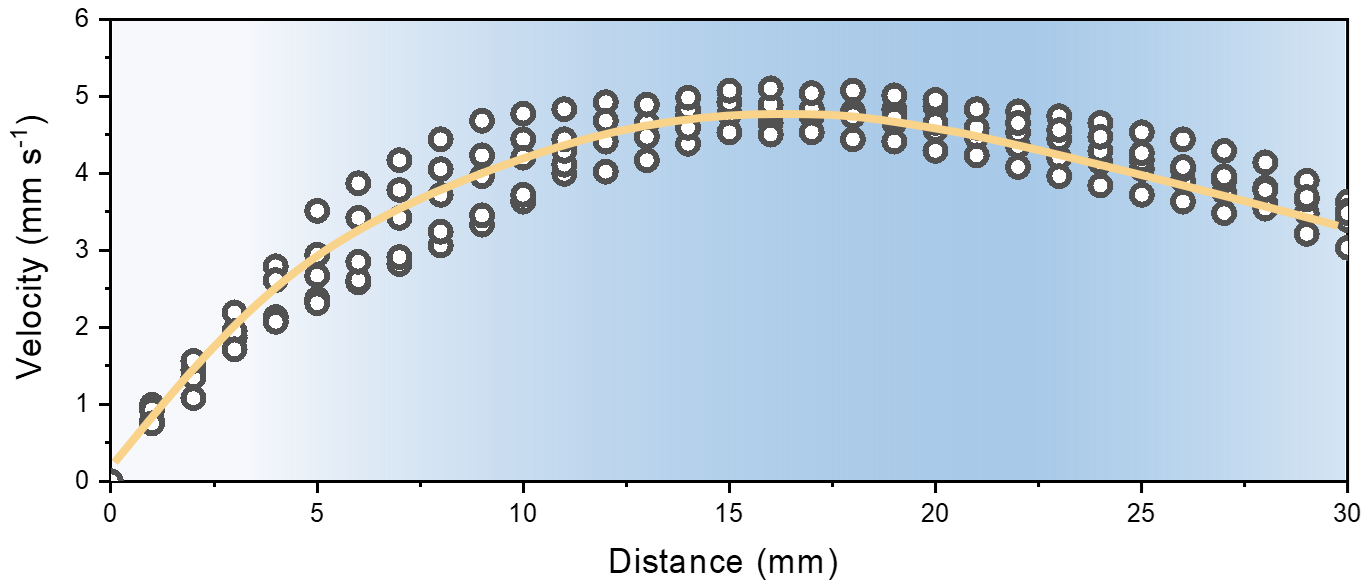


**Fig. S4** The curve of velocity variation with the distance of droplet motion

**Note S3 Dynamics of droplet self-propulsion**

Due to the combined effects of the pyroelectric effect, as well as the charge transfer between the droplet and the lithium niobate (LN) substrate, continuous contact and motion of the supercooled droplet leads to the gradual redistribution of surface potential on the LN (as shown in Fig. S3). Therefore, to elucidate the dynamics of droplet self-propulsion, we calculated the droplet velocity along the trajectory of the droplet motion (Fig. S4). When a supercooled droplet first contacts the SDSS, the temperature variation induces a pyroelectric effect, altering the internal dipole alignment of LN and resulting in charge transfer. The resulting electrostatic repulsion propels the supercooled droplet toward the edge. During this process shown in Fig. R10, as the droplet movement, region of the surface potential gradually expands and the transferred charge accumulates, which causes the increased droplet velocity. However, once the temperature difference between the droplet and the SDSS substrate gradually diminishes (Fig. R9b), the electrostatic repulsive force is reduced, leading to a decrease in the droplet transfer velocity. As a result, the overall self-propulsion velocity of the droplet initially increases, then stabilizes, and finally gradually decreases.


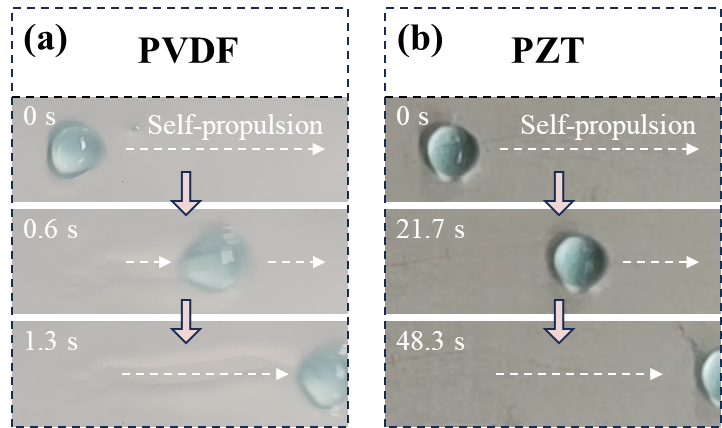


**Fig. S5** Cold droplets (0 °C) self-draining on the slippery surface (27.6 °C) equipped with pyroelectrical materials, no matter in terms of organic PVDF **a**, or inorganic PZT **b**

**
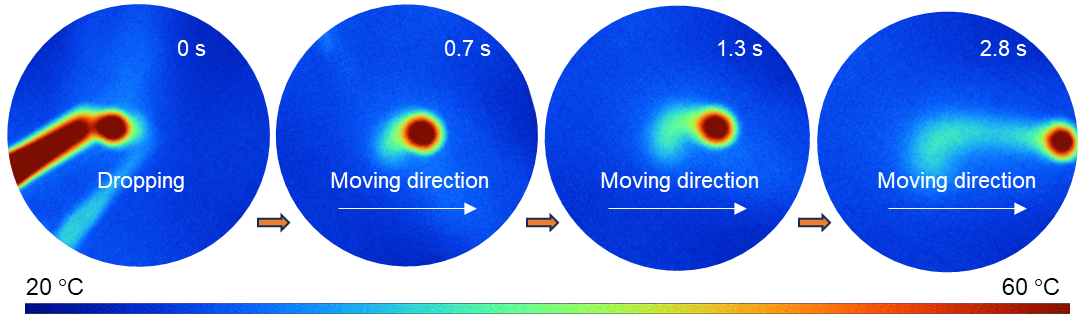
**

**Fig. S6** A series of images illustrating the self-propulsion of high-temperature droplets (60 °C) on the low-temperature SDSS surface (25 °C)

**Note S4 Self-propulsion of hot droplets on SDSS**

The autonomous self-propulsion of droplets plays an indispensable role in high-throughput applications. In addition to anti-icing, large-scale droplet self-transport also has broad utility in fields such as condensation and heat transfer. Although the related experiments and applications are not the main focus of this manuscript, it is noteworthy that the substantial temperature difference between low-temperature condensation devices and high-temperature droplets could facilitate highly efficient mass and heat transfer. To explore this possibility, we characterized the self-propelled behavior of high-temperature droplets on the SDSS surface (Fig. S5). Continuous infrared thermal imaging demonstrates that when high-temperature droplets (60 °C) come into contact with the SDSS surface at room temperature (25 °C), spontaneous removal also occurs. In this case, the de-polarized dipoles within the SDSS more readily form the double electric layers with the hot droplet as it contacts the surface, resulting in the droplet acquiring a significant net negative charge after departure. Therefore, by leveraging the synergistic effects of thermoelectric coupling and charge transfer mechanism, SDSS can serve not only in anti-icing applications but also holds promise for enhancing heat and mass transfer efficiency in thermal management devices, mitigating performance degradation caused by droplet adhesion.


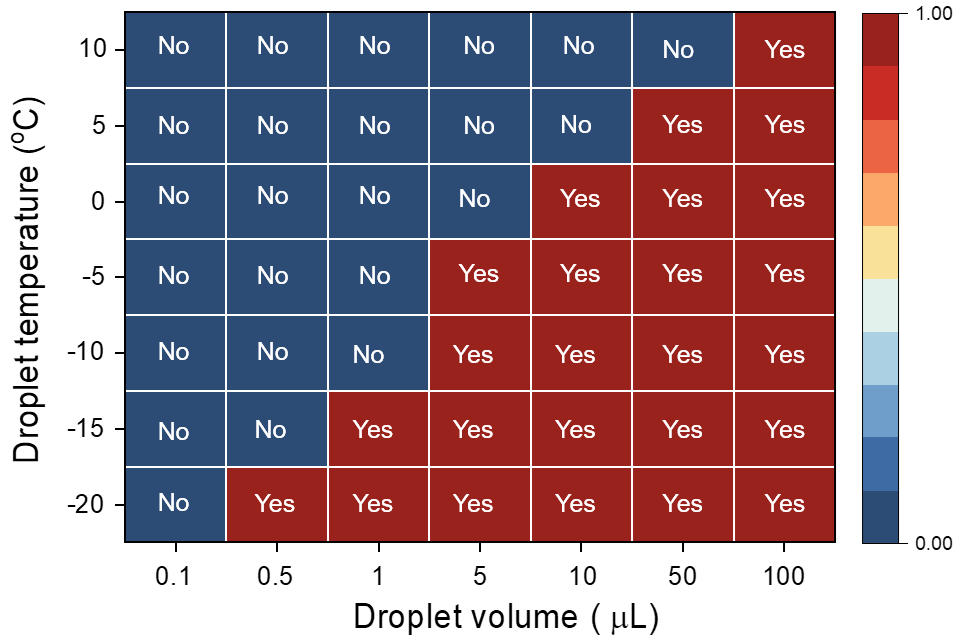


**Fig. S7** A series of images illustrating the self-propulsion of high-temperature droplets (60 °C) on the low-temperature SDSS surface (25 °C)

**
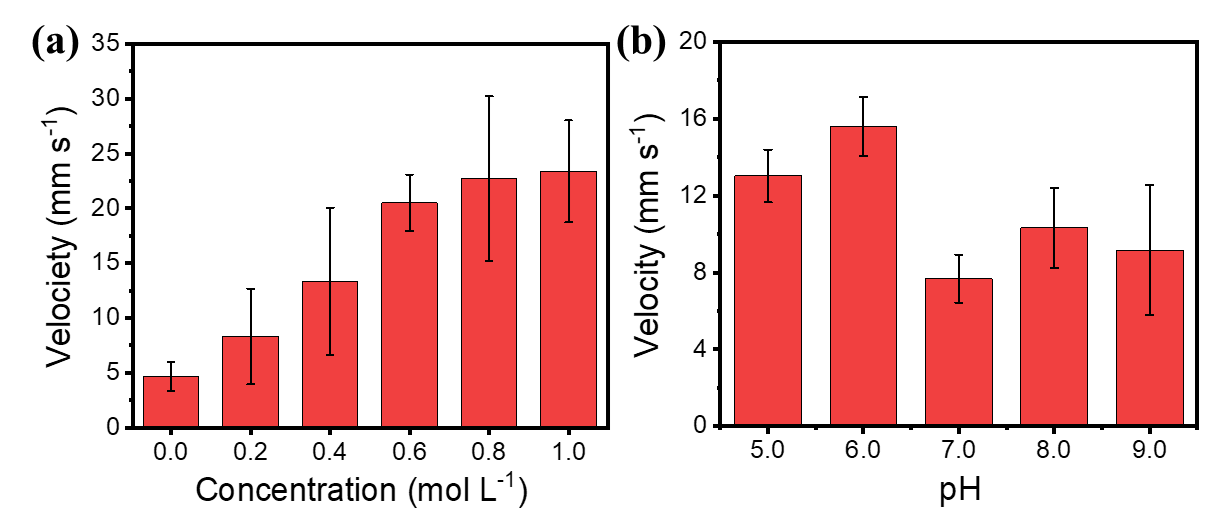
**

**Fig. S8** Droplet velocity as the function of the increased NaCl concentration **a** and pH **b**


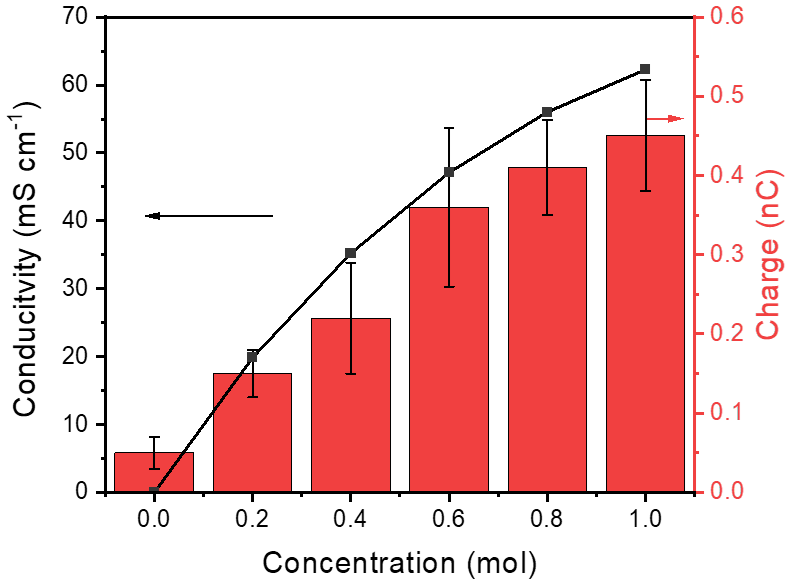


**Fig. S9** The relationship between NaCl concentration, solution conductivity, and transferred charge. Droplets volume, 10 μL; and temperature difference between SDSS and droplets, 20 °C


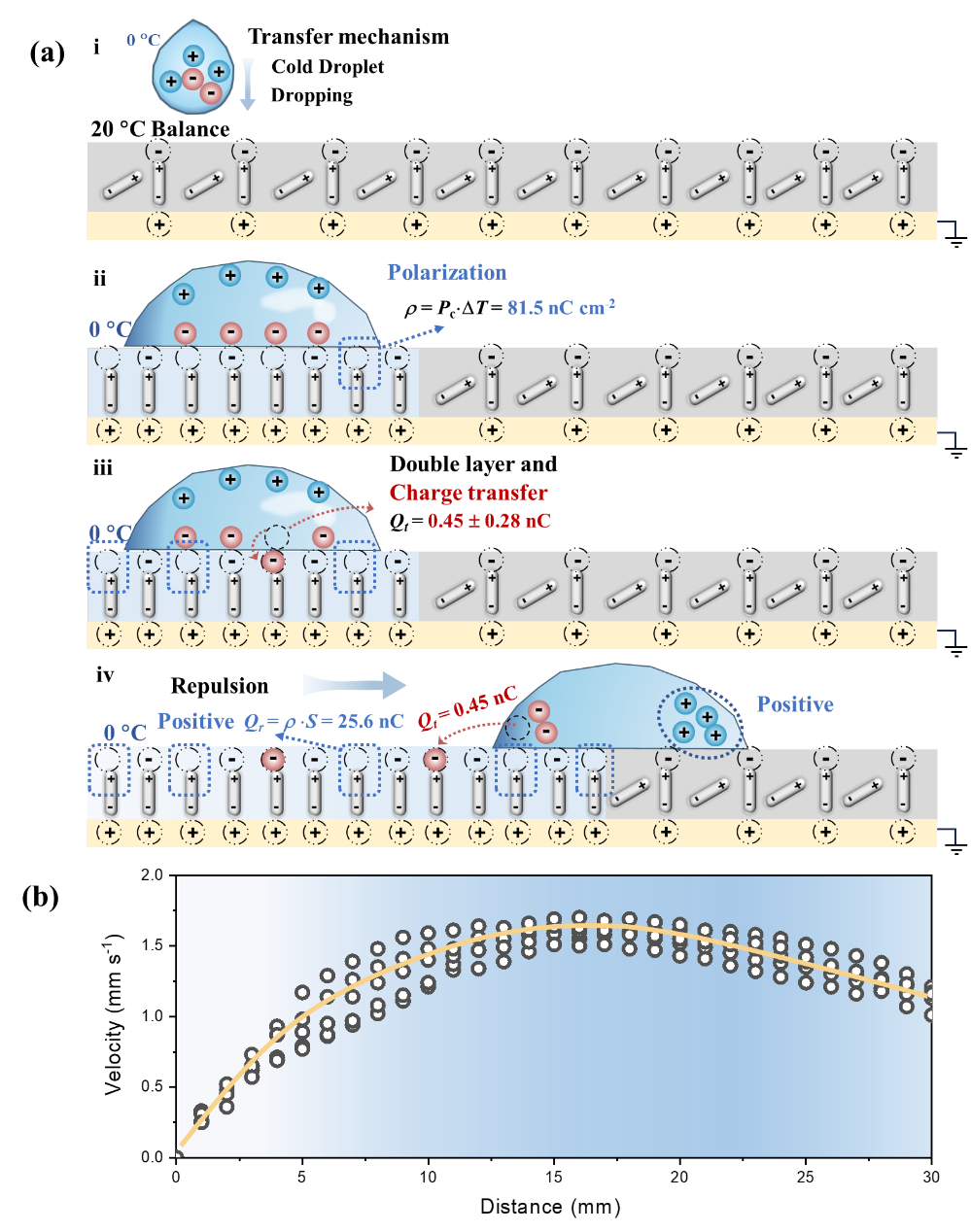


**Fig. S10** Schematic of the mechanism for droplets with ions (NaCl)


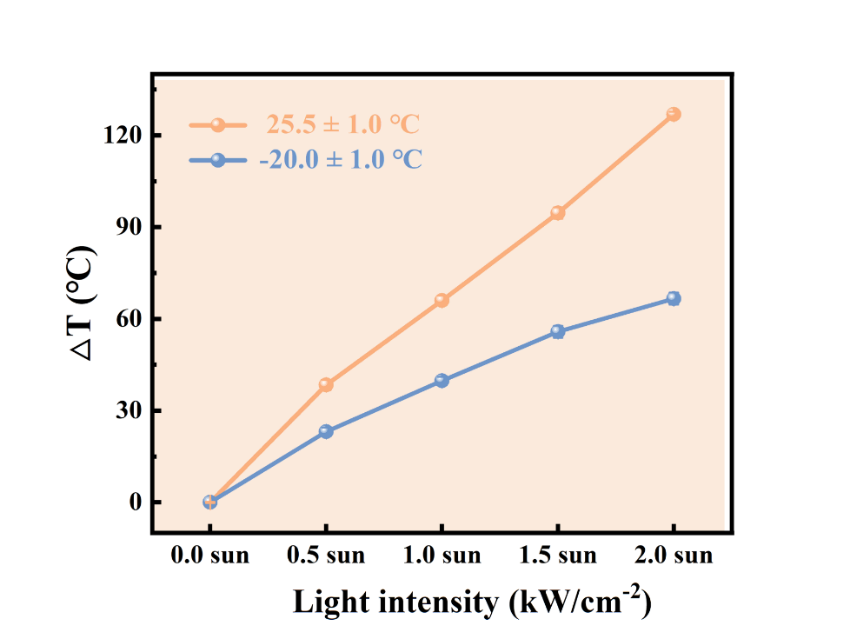


**Fig. S11** The temperature rise (∆T) of the SDSS surface under different light intensity was measured at 25.5 ± 1.0 ℃ and -20.0 ± 1.0 ℃

**
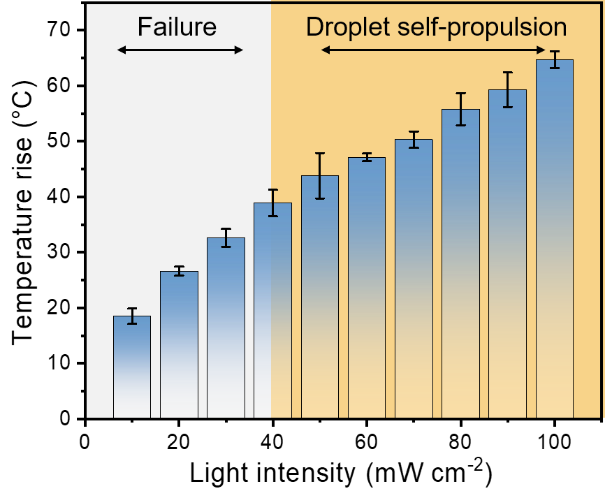
**

**Fig. S12** Relationship between incident light intensity and the temperature rise of the SDSS. When the light intensity exceeds 40 mW cm^-2^, the electrostatic force generated by the temperature difference between SDSS (T > 6 °C) and droplets (10 μL, temperature ~ 25 °C) is sufficient to induce spontaneous movement of droplets on the surface
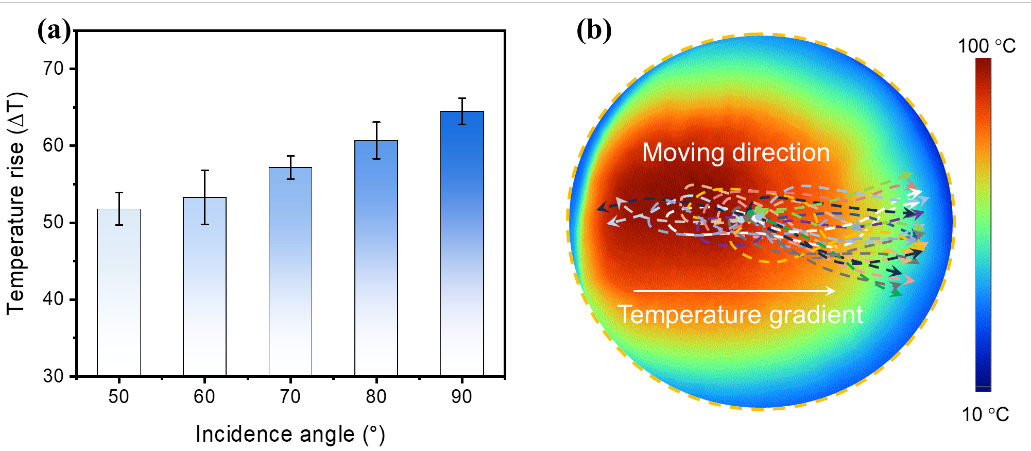


**Fig. S13** **a** Temperature rise of the SDSS vs incidence angle. **b** Surface temperature distribution of the SDSS at a solar incident angle of 60°. The lines shown in panel **b** represent the statistical trajectories of 50 droplets, each released at the center of the SDSS

**Note S5 Self-propulsion of hot droplets on SDSS**

In practical situations, sunlight rarely strikes surfaces perpendicularly. Therefore, we systematically investigated the temperature rise of SDSS and the corresponding droplet dynamic behavior under varying incident angles of sunlight (100 W cm⁻²) ranging from 50° to 90°. As shown in the Fig. R18, as the incident angle decreases, the maximum temperature rise at the center of the SDSS surface gradually drops from 64.5 °C to approximately 51.8 °C (Fig. S13a). Meanwhile, the surface temperature of the SDSS exhibits a gradient distribution, with higher temperatures on the side closer to the light source and lower temperatures on the side further away.

As a result, when droplets are similarly dispensed at the center of the sample, the gradient distribution of both surface temperature and potential leads to anisotropic droplet motion, rather than random, isotropic spreading. By tracking the trajectories of 50 droplets, we observed that when the incident angle is 60°, droplets consistently migrate spontaneously along the direction of temperature gradient, rather than divergent movement. This is likely because the temperature gradient caused by incident angle triggered an asymmetric potential gradient, where the potential near the light source side is higher. Therefore, the dielectrophoretic force induced by potential gradient drive the droplets toward the region with higher potential. During this process, as the surface charge transferred to droplets, the gradually formed electrostatic repulsion surpasses the dielectrophoretic force and becomes the dominant factor, causing the droplets turn to side of the lower potential – the region with a relatively lower temperature. Overall, the temperature and corresponding potential gradients induced by oblique sunlight irradiation facilitate the movement of interfacial droplets toward the edge (Fig. S13b).


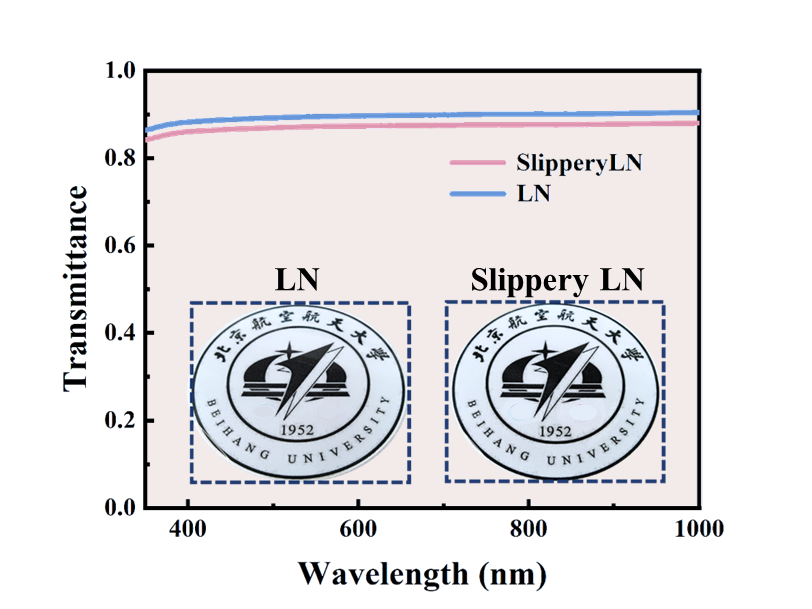


**Fig. S14** The transmittance and optical photo of LN before and after covering the slippery layer

**
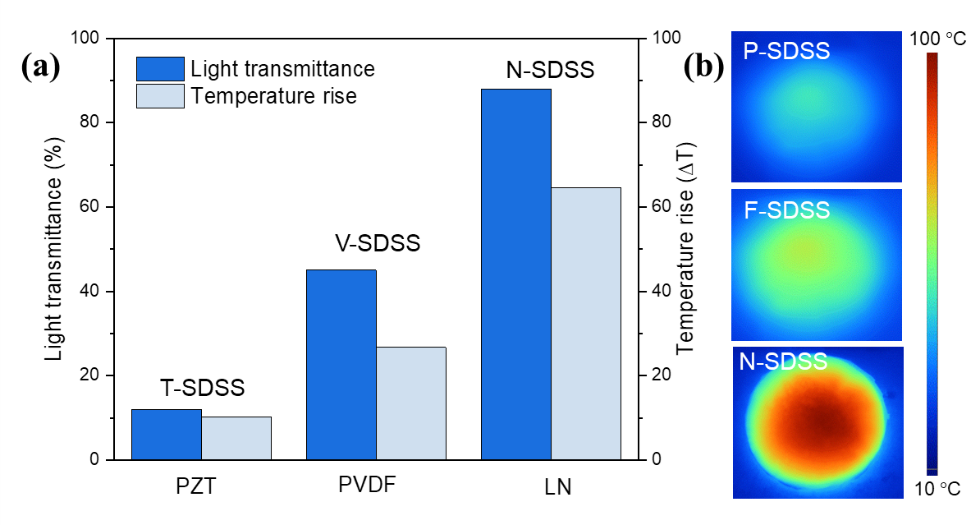
**

**Fig. S15** The temperature rise of SDSS devices with pyroelectric layers of varying transmittance under 1 sun illumination **a**, and the corresponding IR images **b**

**
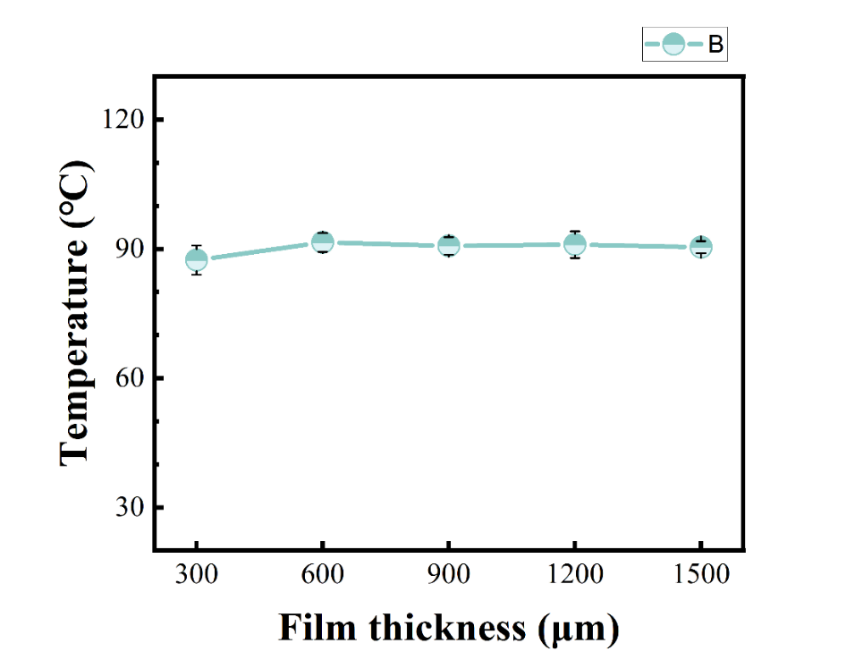
**

**Fig. S16** The surface temperature of 50 wt% CNTs photothermal films with different film thickness under one sunlight


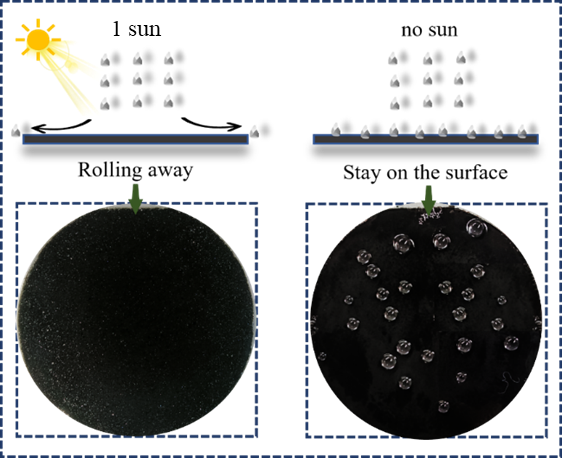


**Fig. S17** Droplets self-removing on the SDSS with assistance of 1 sun, as the comparison of surface with residual droplets without sunlight


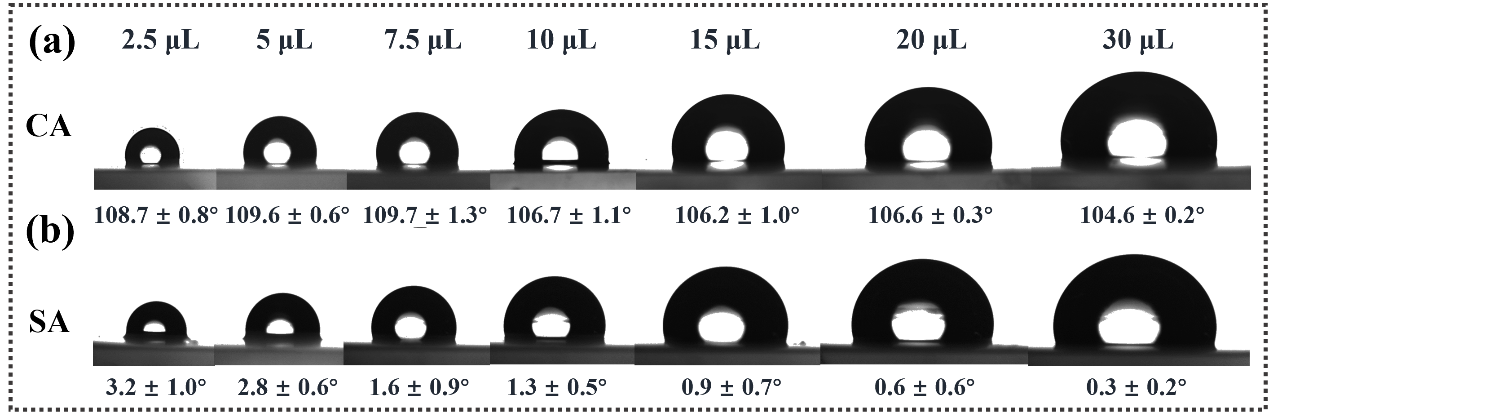


**Fig. S18 a** CAs and **b** SAs of different volume water droplets on SDSS surfac


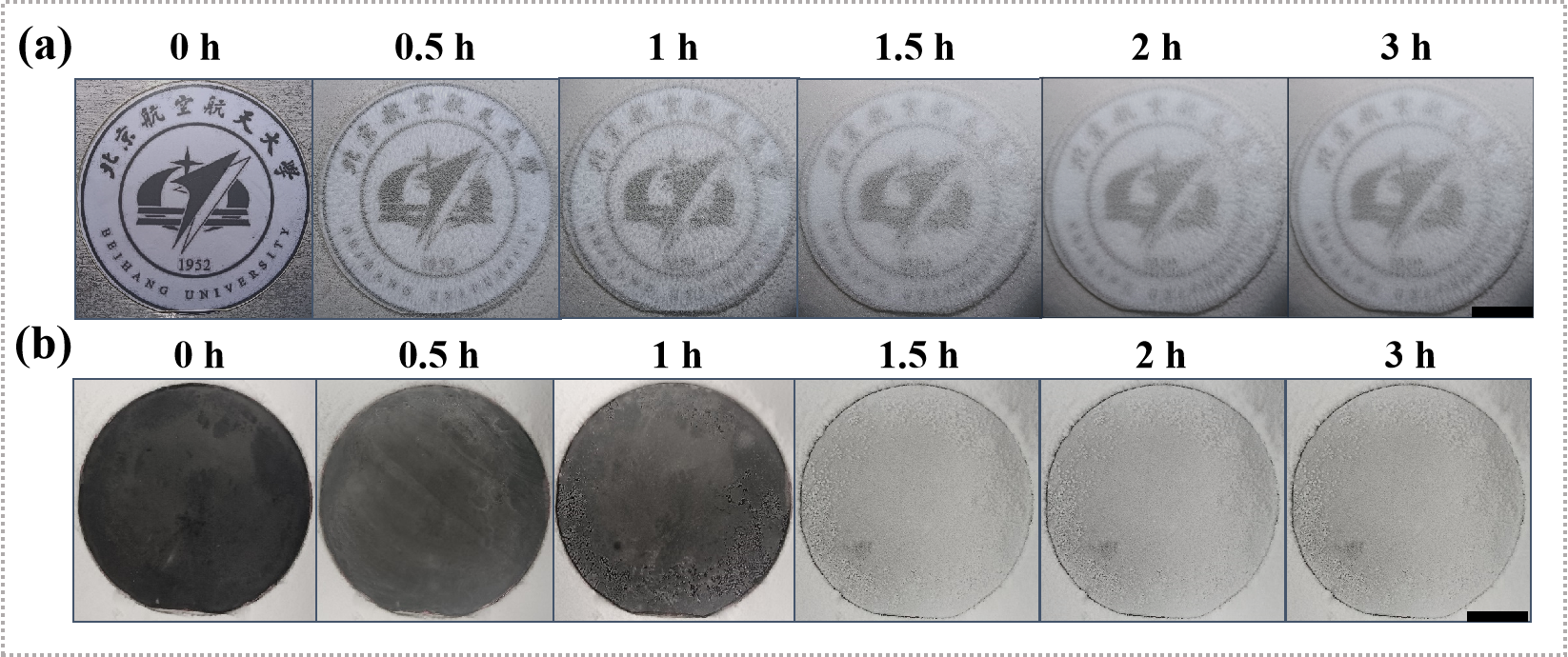


**Fig. S19** The icing of **a** PDMS and **b** TPS without sunlight at -20.0 ± 1.0 ℃ and the relative humidity of 80%. Scale bars are 2 cm


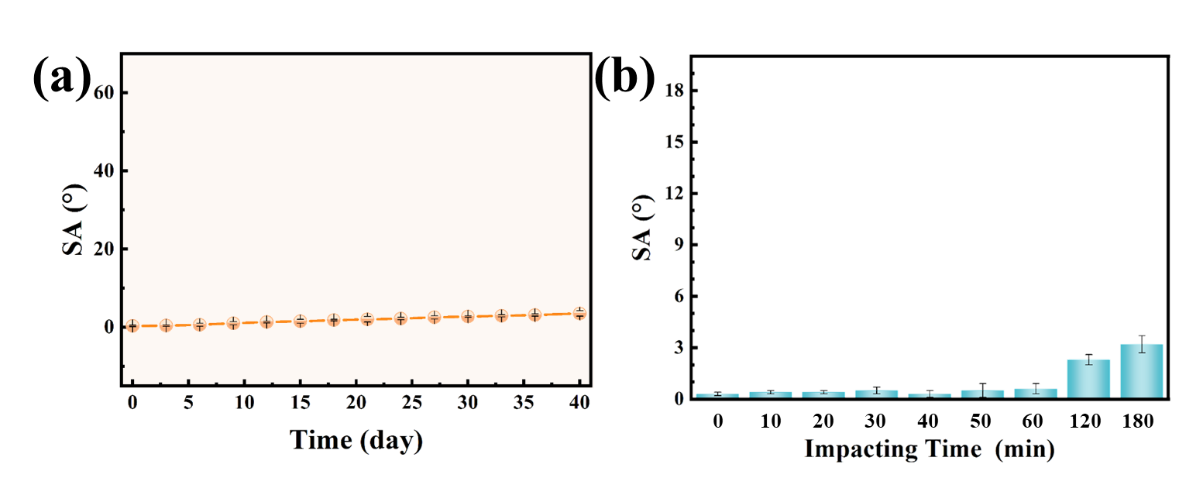


**Fig. S20 a** Variation of water (30 μL) SAs on SDSS in the air (at 80% relative humidity) with stored time. **b** Variation of water (30 μL) SAs on SDSS with the time of water flow impacting


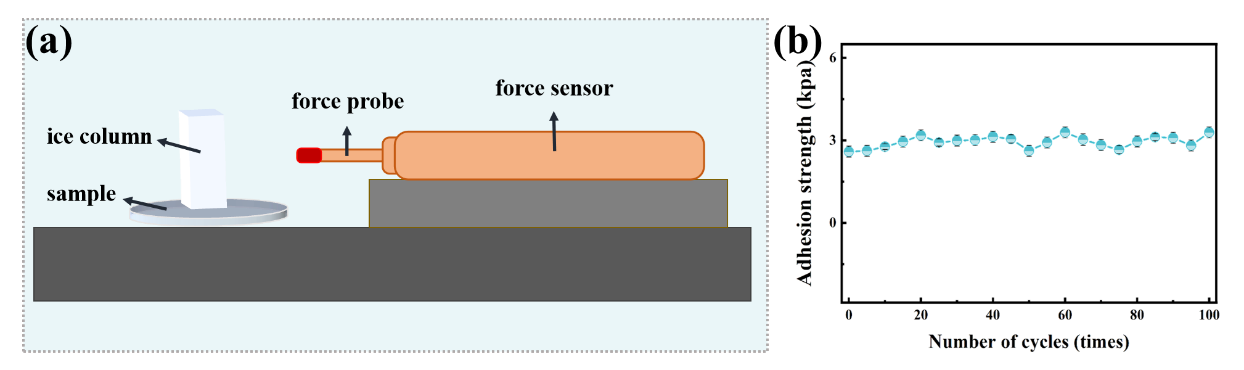


**Fig. S21 a** The device diagram of ice adhesion strength measurement. **b** The adhesion strength changes of ice on SDSS with the number of freezing/thawing cycles
